# Supplementary material for: Computational screen-out strategy for electrically pumped organic laser materials
Source: Nat Commun. 2020 Sep 8;11:4485. doi: 10.1038/s41467-020-18144-x (PMC7478980; doi:10.1038/s41467-020-18144-x)
Supplement: Supplementary file 1 — Supplementary Information [file 41467_2020_18144_MOESM1_ESM.pdf]

Supplementary Information

Computational screen-out strategy for electrically pumped organic laser materials

Ou Q. et al.

Supplementary Tables

Supplementary Table 1. Theoretical and experimental emission energies of DPASBF, αNPD, and CzPAn.

	B3LYP	OT LRC-ωPBE	Exp.	ω value
DPASBF	1.543 eV	2.290 eV	2.254 eV	0.18 a_0^{-1}
αNPD	2.202 eV	2.845 eV	2.786 eV	0.17 a_0^{-1}
CzPAn	2.539 eV	2.672 eV	2.725 eV	0.17 a_0^{-1}

Supplementary Table 2. Theoretical oscillator strength of S1 (f_{em}), reorganization energies (λ_{aps}) and experimental PLQY of investigated materials.

Molecule Group I (experimental single crystal materials)									
**Molecule**	f_{em}	λ_{aps}**(cm^{-1})**	**Exp. PLQY(%)**[a]	**Ref.**	**Molecule**	f_{em}	λ_{aps}**(cm^{-1})**	**Exp. PLQY(%)**	**Ref.**
DSB(2PV)	1.9634	3079	83~87	1	βDBDCS	1.8776	2782	84	2
3PV	2.8179	2739	50~52	1	αMODBDCS	1.4043	2879	42	3
pMDSB	2.1534	3076	87~91	1,4	βMODBDCS	1.5845	2491	46	3
oMDSB	1.9135	3802	88	4	βPDCS	1.343	4068	95	5
4mDSB	2.0408	2815	81	6	βTFDCS	1.7958	3596	58	7
CNMODSB	2.2049	2192	54~62	8	DPA	0.1505	3242	41	9
MSMODSB	1.7664	2362	81	10	DPEA	0.2954	2891	30	11
PDSB	1.7801	3945	76~84	12	TPDSB[c]	2.6677	5627	4~8	13
BMSA	0.8443	4326	35	14	AC5	1.9633	4722	n.a.	15
βDCS	1.6899	3360	n.a[b]	16	BP3T	2.5353	3600	80	17
αMODCS	0.5729	4006	66	3	P6T	2.9818	2755	n.a.	18
βMODCS	0.9726	2541	73	3	TPB	0.8893	3156	30	19
αDBDCS	1.9777	3408	62	20					
Molecule Group II (experimental thin film materials)									
**Molecule**	f_{em}	λ_{aps}**(cm^{-1})**	**Exp. PLQY(%)**	**Ref.**	**Molecule**	f_{em}	λ_{aps}**(cm^{-1})**	**Exp. PLQY(%)**	**Ref.**
CzPSBF	1.6007	3575	55	21	αNPD*[c]	0.1744	2990	27~31	22
CzPVSBF	2.8021	2700	40~44	23	DPABP	0.8603	4099	42~46	22
TSBF	2.2763	4346	70~74	24	TPD	0.8847	4134	39~43	22
TPASBF	1.8734	3584	46	21	BSBCz	2.8766	3193	76	25
DPASBF*	0.0003	4657	27	26	CzPAn*	0.5260	4795	58	27

[a] The values of experimental PLQY are taken from the cited references of undoped materials (either single crystal or thin film) to rule out the influence of the host materials.

[b] n.a.= not available.

[c] Molecules with asterisk are evaluated with an optimal-tuning LRC-ωPBE functional.

Supplementary Table 3. XSAPT+MBD energy decomposition of all investigated π-stacking materials.[a]

**Molecule**	E_{tot}	E_{disp}	E_{elst}	E_{exch}	E_{ind}	$E_{\mathrm{exch-ind}}$
oMDSB	-12.75	-18.71	-5.08	11.73	-3.18	2.49
CNMODSB	-16.16	-25.03	-6.07	16.11	-5.92	4.76
MSMODSB	-17.37	-23.10	-7.98	14.69	-4.97	3.99
BMSA	-19.90	-27.57	-6.60	15.24	-4.18	3.22
αMODCS	-10.93	-21.95	-5.85	16.46	-5.70	5.22

βMODCS	-21.80	-33.38	-8.25	21.24	-6.95	5.54
αDBDCS	-22.05	-30.98	-7.54	17.26	-5.51	4.26
αMODBDCS	-31.81	-51.55	-12.30	34.04	-11.84	9.84
βMODBDCS	-32.24	-56.01	-13.16	38.31	-12.83	11.44
βTFDCS	-19.99	-31.88	-6.84	20.55	-8.21	6.39
TPDSB	-18.64	-26.05	-7.93	16.59	-5.88	4.63
TPB	-17.33	-23.98	-5.87	13.09	-3.09	2.53

[a] All energetics are shown in the unit of KCal mol^{-1}.

Supplementary Table 4. Theoretical and experimental PLQY for twelve electrical pumping candidates.

Molecule	Cal. PLQY (%)[a]	Exp. PLQY (%)[b]
oMDSB	92	88
pMDSB	87	87~91
βDCS	67	n.a.
βDBDCS	89	84
βPDCS	86	95
BP3T	63	80
TPD	43	39~43
CzPSBF	45	55
CzPVSBF	63	40~44
TPASBF	43	46
TSBF	92	70~74
BSBCz	82	76

[a] Theoretical PLQY is evaluated via $\Phi = k_r/(k_r + k_{ic} + k_{isc})$.
[b] Numbers are taken from the same cited references in Supplementary Table 2 of undoped materials (either single crystal or thin film) to rule out the influence of the host materials.

Supplementary Table 5. Transition energy and oscillator strengths near the emission wavelength of twelve candidates.

Molecule	Transition type	Transition energy (eV)	Oscillator strength
oMDSB	$S_0 \rightarrow S_1$	3.217	1.760
	$S_1 \rightarrow S_0$	2.742	1.913
	$S_1 \rightarrow S_n$	2.682	0.105
		3.332	0.055
	$T_1 \rightarrow T_n$	2.202	2.689
	$D_0^+ \rightarrow D_n^+$	2.354	1.557
	$D_0^- \rightarrow D_n^-$	2.342	1.713
pMDSB	$S_0 \rightarrow S_1$	3.163	2.046
	$S_1 \rightarrow S_0$	2.782	2.153
	$S_1 \rightarrow S_n$	2.582	0.125
		3.232	0.055
	$T_1 \rightarrow T_n$	2.207	2.806
	$D_0^+ \rightarrow D_n^+$	2.362	1.724
	$D_0^- \rightarrow D_n^-$	2.376	1.880
βDCS	$S_0 \rightarrow S_1$	3.014	1.537
	$S_1 \rightarrow S_0$	2.597	1.690
	$S_1 \rightarrow S_n$	2.098	0.206
		2.222	0.012
		2.637	0.034
		3.000	0.041
	$T_1 \rightarrow T_n$	2.195	2.196
		2.682	0.075
		3.288	0.063
	$D_0^+ \rightarrow D_n^+$	2.224	1.360
		2.553	0.064
	$D_0^- \rightarrow D_n^-$	2.314	1.551
		2.546	0.078
		2.881	0.023
βDBDCS	$S_0 \rightarrow S_1$	2.750	1.650
	$S_1 \rightarrow S_0$	2.405	1.878

	$S_1 \rightarrow S_n$	2.096	0.168
		2.211	0.079
	$T_1 \rightarrow T_n$	1.986	2.093
		2.597	0.143
		3.027	0.054
	$D_0^+ \rightarrow D_n^+$	2.033	1.555
	$D_0^- \rightarrow D_n^-$	2.215	0.385
		2.307	1.425
βPDCS	$S_0 \rightarrow S_1$	3.056	0.886
	$S_1 \rightarrow S_0$	2.557	1.343
	$S_1 \rightarrow S_n$	2.091	0.196
		2.296	0.034
		2.344	0.046
		2.601	0.050
	$D_0^+ \rightarrow D_n^+$	2.202	0.750
		2.911	0.332
	$D_0^- \rightarrow D_n^-$	2.237	1.101
		2.547	0.179
BP3T	$S_0 \rightarrow S_1$	2.640	2.203
	$S_1 \rightarrow S_0$	2.194	2.535
	$S_1 \rightarrow S_n$	1.719	0.102
		1.756	0.074
	$T_1 \rightarrow T_n$	1.792	2.640
		2.718	0.110
	$D_0^+ \rightarrow D_n^+$	1.719	1.104
		1.971	0.687
	$D_0^- \rightarrow D_n^-$	1.748	0.525
		2.640	1.140
TPD	$S_0 \rightarrow S_1$	3.414	1.102
	$S_1 \rightarrow S_0$	2.901	0.885
	$S_1 \rightarrow S_n$	2.853	0.067
	$T_1 \rightarrow T_n$	2.786	0.058
		3.100	0.101
		3.437	0.075
	$D_0^+ \rightarrow D_n^+$	2.741	0.067
		2.953	0.712
	$D_0^- \rightarrow D_n^-$	3.192	0.923
		3.461	0.118
CzPSBF	$S_0 \rightarrow S_1$	3.342	1.122
	$S_1 \rightarrow S_0$	2.872	1.601
	$T_1 \rightarrow T_n$	2.225	0.260
		2.390	0.080
		2.561	0.057
	$D_0^+ \rightarrow D_n^+$	3.221	0.242
		3.382	0.142
	$D_0^- \rightarrow D_n^-$	2.753	1.286
CzPVSBF	$S_0 \rightarrow S_1$	2.784	2.735
	$S_1 \rightarrow S_0$	2.449	2.802
	$S_1 \rightarrow S_n$	1.863	0.109
	$T_1 \rightarrow T_n$	1.897	0.754
		2.497	0.134
		2.855	0.169
	$D_0^+ \rightarrow D_n^+$	2.058	0.750
		2.634	0.271
		2.817	0.080
		2.897	0.270
	$D_0^- \rightarrow D_n^-$	2.190	1.891
		2.896	0.050
TPASBF	$S_0 \rightarrow S_1$	3.173	1.732
	$S_1 \rightarrow S_0$	2.709	1.873
	$S_1 \rightarrow S_n$	2.284	0.026
		2.292	0.015
		2.304	0.065

		2.313	0.049
	$T_1 \rightarrow T_n$	2.227	0.081
		2.430	0.100
		2.730	0.056
	$D_0^+ \rightarrow D_n^+$	2.611	0.667
		3.153	0.066
		3.428	0.130
		3.486	0.398
	$D_0^- \rightarrow D_n^-$	2.696	1.326
TSBF	$S_0 \rightarrow S_1$	3.373	1.999
	$S_1 \rightarrow S_0$	2.834	2.276
	$T_1 \rightarrow T_n$	2.305	0.120
	$D_0^+ \rightarrow D_n^+$	2.610	0.751
		3.056	0.047
		3.215	0.235
	$D_0^- \rightarrow D_n^-$	2.551	1.130
BSBCz	$S_0 \rightarrow S_1$	2.909	2.199
	$S_1 \rightarrow S_0$	2.472	2.877
	$S_1 \rightarrow S_n$	1.808	0.128
	$T_1 \rightarrow T_n$	1.893	0.810
		2.420	0.109
		2.806	0.093
	$D_0^+ \rightarrow D_n^+$	2.112	0.583
		2.781	0.254
		2.923	0.702
	$D_0^- \rightarrow D_n^-$	2.228	1.790
		2.850	0.089
		3.12	0.180

Supplementary Table 6. Calculated SOC and adiabatic energy gap between T_1 and S_0 for twelve candidates.

Molecule	T_1/S_0 SOC (cm^{-1})	T_1/S_0 E_{adia} (eV)
oMDSB	0.0000	1.431
pMDSB	0.0000	1.449
βDCS	0.0038	1.319
βDBDCS	0.0014	1.249
βPDCS	0.0000	1.395
BP3T	0.1355	1.195
TPD	0.0796	2.254
CzPSBF	0.7469	2.234
CzPVSBF	0.4924	1.572
TPASBF	0.7108	2.132
TSBF	0.1662	2.181
BSBCz	0.2577	1.632

Supplementary Table 7. Comparison of QM/MM and single-molecule excitation energies for singlets and triplets.[a]

Excited state #	Singlets		Triplets	
	QM/MM	Single molecule	QM/MM	Single molecule
1	2.447	2.402	1.266	1.221
2	2.972	2.936	2.146	2.110
3	3.708	3.650	2.979	2.912
4	3.813	3.788	3.114	3.105
5	3.879	3.850	3.359	3.346
6	3.902	3.872	3.554	3.543
7	3.940	3.922	3.597	3.561
8	4.342	4.301	3.626	3.618
9	4.507	4.501	3.663	3.622

10	4.548	4.523	3.928	3.906
11	4.608	4.616	3.933	3.920
12	4.664	4.659	4.095	4.080
13	5.033	4.956	4.169	4.205
14	5.062	5.031	4.201	4.220
15	5.079	5.096	4.431	4.438
16	5.170	5.143	4.655	4.652
17	5.204	5.188	4.673	4.680
18	5.225	5.219	4.744	4.683
19	5.240	5.230	4.774	4.701
20	5.274	5.251	4.866	4.859

[a] The lowest 20 singlet and triplet excitation energies of βDBDCS are obtained from QM/MM and single molecule calculations at S_1 optimized geometry and T_1 optimized geometry, respectively. All values are shown in the unit of eV. Derivations between QM/MM and single molecule calculations are within 0.1 eV.

Supplementary Table 8. Comparison of QM/MM and single-molecule excitation energies for cation and anion.[a]

Excited state #	Cation		Anion	
	QM/MM	Single molecule	QM/MM	Single molecule
1	0.977	1.002	1.533	1.514
2	1.725	1.793	2.164	2.201
3	1.904	1.981	2.180	2.215
4	1.920	1.999	2.301	2.307
5	1.996	2.033	2.606	2.636
6	2.084	2.054	2.672	2.698
7	2.188	2.149	2.824	2.868
8	2.397	2.484	2.846	2.906
9	3.021	2.980	3.388	3.348
10	3.190	3.173	3.593	3.583

[a] The lowest 10 excitation energies of βDBDCS are obtained from QM/MM and single molecule calculations for cation and anion at the corresponding optimized ground state geometries. All values are shown in the unit of eV. Derivations between QM/MM and single molecule calculations are within 0.1 eV.

Supplementary Figures

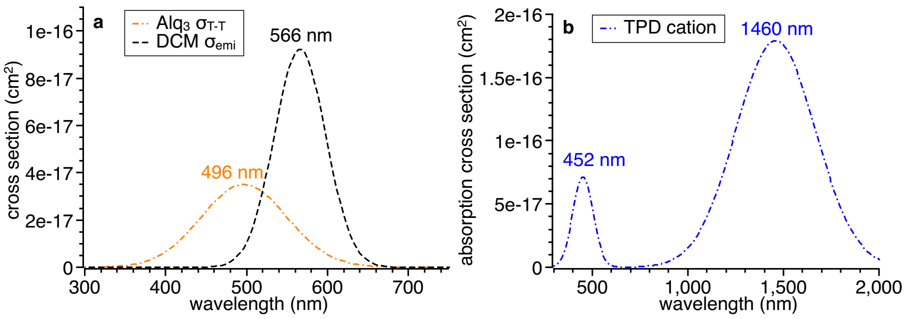

Supplementary Figure 1. Theoretically predicted absorption cross sections for various systems. (**a**) Theoretically predicted triplet-triplet absorption cross section of Alq_3 in benzene solution (green dot-dashed line) and stimulated emission cross section of DCM (black dashed line), and (**b**) theoretically predicted cation absorption cross section of TPD in CH_2Cl_2 solution. Note that a broadening with a 500 nm FWHM is applied to the second absorption maximum of TPD cation due to its extremely low energy, while a normal 125 nm FWHM is applied to the first maximum. For both Alq_3 and TPD, solvent effects are addressed via linear-response polarizable continuum model (LR-PCM).[28–30]

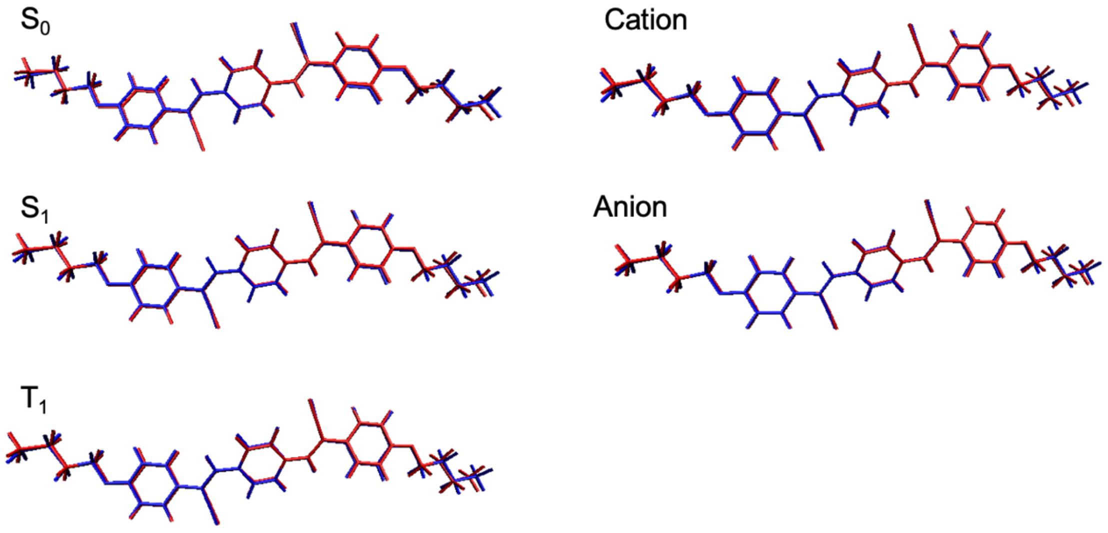

Supplementary Figure 2. Various optimized structures of βDBDCS obtained from QM/MM (red) and single molecule (blue) calculations. Small derivations are mainly observed on alkyl groups, which do not contribute to the electronic excitation.

Reference:

1. Nakanotani, H., Saito, M., Nakamura, H. & Adachi, C. Highly balanced ambipolar mobilities with intense electroluminescence in field-effect transistors based on organic single crystal oligo(p-phenylenevinylene) derivatives. *Appl. Phys. Lett.* **95**, 033308 (2009).
2. Varghese, S. *et al.* Orthogonal Resonator Modes and Low Lasing Threshold in Highly Emissive Distyrylbenzene-Based Molecular Crystals. *Adv. Opt. Mater.* **2**, 542–548 (2014).
3. Yoon, S.-J. *et al.* Color-Tuned, Highly Emissive Dicyanodistyrylbenzene Single Crystals: Manipulating Intermolecular Stacking Interactions for Spontaneous and Stimulated Emission Characteristics. *Adv. Opt. Mater.* **1**, 232–237 (2013).
4. Kabe, R., Nakanotani, H., Sakanoue, T., Yahiro, M. & Adachi, C. Effect of Molecular Morphology on Amplified Spontaneous Emission of Bis-Styrylbenzene Derivatives. *Adv. Mater.* **21**, 4034–4038 (2009).
5. Wang, H. *et al.* Cyano-Substituted Oligo(p-phenylene vinylene) Single Crystals: A Promising Laser Material. *Adv. Funct. Mater.* **21**, 3770–3777 (2011).
6. Varghese, S. *et al.* Stimulated Emission Properties of Sterically Modified Distyrylbenzene-Based H-Aggregate Single Crystals. *J. Phys. Chem. Lett.* **4**, 1597–1602 (2013).
7. Varghese, S. *et al.* Polymorphism and Amplified Spontaneous Emission in a Dicyano-Distyrylbenzene Derivative with Multiple Trifluoromethyl Substituents: Intermolecular Interactions in Play. *Adv. Funct. Mater.* **26**, 2349–2356 (2016).
8. Liao, Q. *et al.* An Organic Microlaser Array Based on a Lateral Microcavity of a Single J-aggregation Microbelt. *Angew. Chem. Int. Ed.* **54**, 7037–7041 (2015).
9. Liu, J. *et al.* High mobility emissive organic semiconductor. *Nat. Commun.* **6**, 10032 (2015).
10. Xu, Z. *et al.* Low-Threshold Nanolasers Based on Slab-Nanocrystals of H-Aggregated Organic Semiconductors. *Adv. Mater.* **24**, OP216–OP220 (2012).
11. Liu, D. *et al.* A new organic compound of 2-(2,2-diphenylethenyl)anthracene (DPEA) showing simultaneous electrical charge transport property and AIE optical characteristics. *J. Mater. Chem. C* **6**, 3856–3860 (2018).
12. Xie, Z. *et al.* Crystal Structure of a Highly Luminescent Slice Crystal Grown in the Vapor Phase: A New Polymorph of 2,5-Diphenyl-1,4-distyrylbenzene. *Cryst. Growth Des.* **7**, 2512–2516 (2007).
13. Liao, Q. *et al.* The effect of 1D- and 2D-polymorphs on organic single-crystal optoelectronic devices: lasers and field effect transistors. *J. Mater. Chem. C* **6**, 7994–8002 (2018).
14. Xu, B. *et al.* Solid state emission enhancement of 9,10-distyrylanthracene derivatives and amplified spontaneous emission from a large single crystal. *New J. Chem.* **34**, 1838–1842 (2010).
15. Ichikawa, M. *et al.* Improved Crystal-Growth and Emission Gain-Narrowing of Thiophene/Phenylene Co-Oligomers. *Adv. Mater.* **15**, 213–217 (2003).
16. Xu, Y. *et al.* Supramolecular interaction-induced self-assembly of organic molecules into ultra-long tubular crystals with wave guiding and amplified spontaneous emission. *J. Mater. Chem.* **22**, 1592–1597 (2012).
17. Nakanotani, H. & Adachi, C. Amplified Spontaneous Emission and Electroluminescence from Thiophene/Phenylene Co-Oligomer-Doped p-bis(p-Styrylstyryl)Benzene Crystals. *Adv. Opt. Mater.* **1**, 422–427 (2013).
18. Ichikawa, M. *et al.* Laser Oscillation in Monolithic Molecular Single Crystals. *Adv. Mater.* **17**, 2073–2077 (2005).
19. Tavazzi, S. *et al.* Polarized Absorption, Spontaneous and Stimulated Blue Light Emission of J-type Tetraphenylbutadiene Monocrystals. *ChemPhysChem* **11**, 429–434 (2010).

20. Yoon, S.-J. *et al.* Multistimuli Two-Color Luminescence Switching via Different Slip-Stacking of Highly Fluorescent Molecular Sheets. *J. Am. Chem. Soc.* **132**, 13675–13683 (2010).
21. Komino, T., Nomura, H., Yahiro, M., Endo, K. & Adachi, C. Dependence of the Amplified Spontaneous Emission Threshold in Spirofluorene Thin Films on Molecular Orientation. *J. Phys. Chem. C* **115**, 19890–19896 (2011).
22. Kawamura, Y. *et al.* Ultraviolet amplified spontaneous emission from thin films of 4,4′-bis(9-carbazolyl)-2,2′-biphenyl and the derivatives. *Appl. Phys. Lett.* **84**, 2724–2726 (2004).
23. Nakanotani, H. *et al.* Extremely Low-Threshold Amplified Spontaneous Emission of 9,9′-Spirobifluorene Derivatives and Electroluminescence from Field-Effect Transistor Structure. *Adv. Funct. Mater.* **17**, 2328–2335 (2007).
24. Oyamada, T. *et al.* Optical Properties of Oligo(9,9-diarylfluorene) Derivatives in Thin Films and Their Application for Organic Light-Emitting Field-Effect Transistors. *J. Phys. Chem. C* **111**, 108–115 (2007).
25. Sandanayaka, A. S. D. *et al.* Quasi-Continuous-Wave Organic Thin-Film Distributed Feedback Laser. *Adv. Opt. Mater.* **4**, 834–839 (2016).
26. Nakagawa, T., Ku, S.-Y., Wong, K.-T. & Adachi, C. Electroluminescence based on thermally activated delayed fluorescence generated by a spirobifluorene donor–acceptor structure. *Chem. Commun.* **48**, 9580–9582 (2012).
27. Niu, Q. *et al.* Solution-processed anthracene-based molecular glasses as stable blue-light-emission laser gain media. *Org. Electron.* **18**, 95–100 (2015).
28. Miertuš, S., Scrocco, E. & Tomasi, J. Electrostatic interaction of a solute with a continuum. A direct utilizaion of AB initio molecular potentials for the prevision of solvent effects. *Chem. Phys.* **55**, 117–129 (1981).
29. Cammi, R. & Mennucci, B. Linear response theory for the polarizable continuum model. *J. Chem. Phys.* **110**, 9877–9886 (1999).
30. Cossi, M. & Barone, V. Time-dependent density functional theory for molecules in liquid solutions. *J. Chem. Phys.* **115**, 4708–4717 (2001).
